# Impact on total population health and societal cost, and the implication on the actual cost-effectiveness of including tumour necrosis factor-α antagonists in management of ankylosing spondylitis: a dynamic population modelling study

**DOI:** 10.1186/s12962-015-0044-x

**Published:** 2015-10-07

**Authors:** An Tran-Duy, Annelies Boonen, Mart A. F. J. van de Laar, Johan L. Severens

**Affiliations:** Department of Clinical Epidemiology and Medical Technology Assessment, Maastricht University Medical Center, P.O. Box 5800, 6202 AZ Maastricht, The Netherlands; Division of Rheumatology, Department of Internal Medicine, Maastricht University Medical Center, Maastricht, The Netherlands; Julius Center for Health Sciences and Primary Care, University Medical Center Utrecht, Utrecht, The Netherlands; Caphri School for Public Health and Primary Care, Maastricht UMC+, Maastricht, The Netherlands; Department of Rheumatology and Clinical Immunology, Twente University and Medisch Spectrum Twente, Enschede, The Netherlands; Institute of Health Policy and Management, Erasmus University Rotterdam, Rotterdam, The Netherlands

**Keywords:** Budget impact, Health impact, Cost-effectiveness, Cost-utility, Discrete event simulation, Microsimulation, Modelling, Population dynamics, Tumor necrosis factor, Ankylosing spondylitis

## Abstract

**Background:**

Sequential treatment of ankylosing spondylitis (AS) that includes tumour necrosis factor-α antagonists (anti-TNF agents) has been applied in most of the Western countries. Existing cost-effectiveness (CE) models almost exclusively presented the incremental CE of anti-TNF agents using a closed cohort while budget impact studies are mainly lacking. Notwithstanding, information on impact on total population health and societal budget as well as on actual incremental CE for a given decision time span are important for decision makers. This study aimed at quantifying, for different decision time spans starting from January 1, 2014 in the Dutch society, (1) impact of sequential drug treatment strategies without and with inclusion of anti-TNF agents (Strategies 1 and 2, respectively) on total population health and societal cost, and (2) the actual incremental CE of Strategy 2 compared to Strategy 1.

**Methods:**

Dynamic population modelling was used to capture total population health and cost, and the actual incremental CE. Distinguishing the prevalent AS population on January 1, 2014 and the incident AS cohorts in the subsequent 20 years, the model tracked individually an actual number of AS patients until death or end of the simulation time. During the simulation, data on patient characteristics, history of drug use, costs and health at discrete time points were generated. In Strategy 1, five nonsteroidal anti-inflammatory drugs (NSAIDs) were available but anti-TNF agents withdrawn. In Strategy 2, five NSAIDs and two anti-TNF agents continued to be available.

**Results:**

The predicted size of the prevalent AS population in the Dutch society varied within the range of 67,145–69,957 with 44–46 % of the patients receiving anti-TNF agents over the period 2014–2034. The use of anti-TNF agents resulted in an increase in the annual drug costs (168.54–205.28 million Euros), but at the same time caused a decrease in the annual productivity costs (12.58–31.21 million Euros) and in annual costs of healthcare categories other than drugs (7.23–11.90 million Euros). Incremental cost (Euros) per QALY gained in Strategy 2 compared to Strategy 1 corresponding to decision time spans of 5, 10, 15 and 20 years improved slightly from 75,379 to 67,268, 63,938 and 61,129, respectively. At willingness-to-pay thresholds of 118,656, 112,067, 110,188 and 110,512 Euros, it was 99 % certain that Strategy 2 was cost-effective for decision time spans of 5, 10, 15 and 20, respectively.

**Conclusions:**

Using the dynamic population approach, the present model can project real-time data to inform a healthcare system decision that affects all actual number of AS patients eligible for anti-TNF agents within different decision time spans. The predicted total population costs of different categories in the present study can help plan the organization of the healthcare resources based on the national budget for the disease.

## Background

Ankylosing spondylitis (AS) is a chronic inflammatory rheumatic disease that imposes a considerable burden to the society [1, 2]. Main characteristics of AS include inflammation of the sacroiliac joints and the spine, which results in back pain and stiffness. The early onset of AS, mostly in the second and third decade of life, contributes to long-term functional impairments and a decrease in quality of life of the patients. Productivity loss due to sick leave and work disability in patients with AS has been found to be substantial [3]. Unfortunately, there is currently no cure for AS. Treatments of AS, conventionally restricted to nonsteroidal anti-inflammatory drugs (NSAIDs) and physiotherapy, only relieve pain and stiffness without slowing down the disease progression [4]. Based on the evidence of large beneficial effects of tumour necrosis factor-α antagonists (anti-TNF agents) on symptoms of AS [5–8], these drugs have been approved for use in most of the Western countries from 2002 onwards. In most guidelines for management of AS, treatment of AS is characterised by a sequence of drugs, in which the first anti-TNF agent is given to patients with persistently high disease activity despite adequate treatments with two or more NSAIDs [9–12]. However, treatment with anti-TNF agents is costly and places much pressure on the healthcare budget [13]. In societies that take into account economic consequences of new technologies, decisions on the coverage and reimbursement are based not only on the cost-effectiveness (CE) data, which represent the economic efficiency of the technologies, but also on the total cost of adopting the technologies and the national healthcare budgets, which are used to assess the technology affordability [14]. Thus, the overall health and budget impacts for a society are increasingly receiving attention from the policy makers [15–18].

To capture the impact of inclusion of anti-TNF agents in the treatment strategy on total population health and cost over a specific period of time, the actual number of patients receiving anti-TNF agents during that period must be determined. This requires a dynamic population approach, that is, new incident cases of AS as well as the AS patients leaving the population over time due to death should be taken into consideration. Also, changes over time in the number of AS patients who are eligible for anti-TNF agents need to be quantified. Using this approach, the actual incremental CE, i.e. incremental CE based on the total population cost and health over a real-time period, can also be computed, which would be useful to inform a decision that affects all actual patients in the healthcare system over a series of relevant time spans determined by a decision maker.

During the past decade, the incremental CE of therapies with anti-TNF agents compared with the usual care has been investigated by several conventional modelling studies [13, 19, 20]. However, no published studies have attempted to predict the impact on total population health and societal cost as well as the actual incremental CE of the inclusion of anti-TNF agents in treatment of AS. One of the challenges in this prediction is to capture the dynamics of the AS population and the actual use of anti-TNF agents in the whole society. In a previous study [20], we developed a discrete event modelling framework to predict the incremental CE of a sequential treatment strategy including anti-TNF agents compared to usual care. Although this model took into consideration the eligibility of a patient for an anti-TNF agent therapy, it did not account for new incident patients entering the simulation over time, and thus cannot be used to compute total population health and cost.

This study aimed at quantifying, for different time spans of decision within the period from January 1, 2014 to January 1, 2034 in the Dutch society, (1) impact of sequential drug treatment strategies without and with inclusion of anti-TNF agents on total population health and societal cost, and (2) the actual incremental CE of inclusion of anti-TNF agents compared to usual care.

## Methods

### General modelling approach and treatment strategies

We developed a dynamic population model to simulate the impact of sequential treatment strategies without and with inclusion of anti-TNF agents on health and costs of all individual patients. The dynamics of the AS population are characterised by real-life changes over time in its size (epidemiological aspect) and in the distributions of the characteristics of the patients (demographic and health aspects) and the distributions of the costs they incurred. These distributions are hereafter referred to as AS population properties. Changes in the size of the AS population are determined by the size of the initial prevalent AS population, the incident cases joining the AS population over time, and the mortality of the patients. The incident cases represent newly diagnosed AS patients who come under care of the rheumatologists. The AS population properties can be captured by simulating changes in characteristics and costs of individual patients. To track the patients individually, the patient-level model (PLMod) developed by Tran-Duy et al. [20] was adapted and used in the present study. PLMod is a discrete event simulation model with three interrelated components, namely entity, states and events. An entity in PLMod is a patient characterized by age, gender, symptom duration, diagnosis duration, work status (employed and work disabled), length of sick leave in one employed, disease activity (Bath AS Disease Activity Index, BASDAI [21]; scaled from 0 to 10 with higher values indicating worse disease activity), and functional status (Bath AS Functional Index, BASFI [22]; scaled from 0 to 10 with higher values indicating worse physical functioning). These characteristics were used to predict health utility, productivity costs and different categories of resource utilization. States in PLMod indicate whether a patient is receiving an NSAID, an anti-TNF agent or palliative care, or indicate whether BASDAI is decreasing, stable or increasing. These states were used to simulate the rheumatologist decision on the next treatment when the patient did not respond sufficiently to the current treatment, or to determine possible events that may occur. Events in PLMod include BASDAI decrease or increase, loss of response to the current treatment, visit to a rheumatologist, occurrence of drug toxicity, selection and start of a new treatment, and death. During the simulation, times to competing events were drawn from appropriate probability distributions and the patient “jumps” to the event with the shortest sampled time. Following the occurrence of an event, states and characteristics of the simulated patient were updated, based on which a next event was predicted. In brief, the simulation in PLMod starts with creation of a virtual patient with characteristics sampled from appropriate distributions; then, PLMod simulates changes in BASDAI and BASFI of the patient under a specific treatment strategy and generates data on other characteristics, treatments, resource utilization and quality adjusted life years (QALYs) at discrete time points until death or end of the simulation time. Extensive descriptions of the model structure, statistical models, mathematical relationships and the simulation process can be found in the study by Tran-Duy et al. [20] (main text and Web Only Data). As in PLMod, we used the same statistical models to link changes in BASDAI to changes in BASFI, and to predict costs and QALYs using BASDAI, BASFI, age, gender, symptom duration and diagnosis duration as explanatory variables (for modelling methods and parameter estimates, see Tran-Duy et al. [20], Web Only Data Appendix 3: Disease progression, resource utilization and health utility estimation). In the present model costs were aggregated into three main categories, which were drug cost, medical cost other than drug cost (including costs of hospitalisation and rehabilitation; visits to rheumatologists and other specialists, to general practitioners, and to nurse specialists, physiotherapists and psychotherapists; and formal and informal care), and productivity cost (including sick leave and work disability costs).

**Fig. 1:**
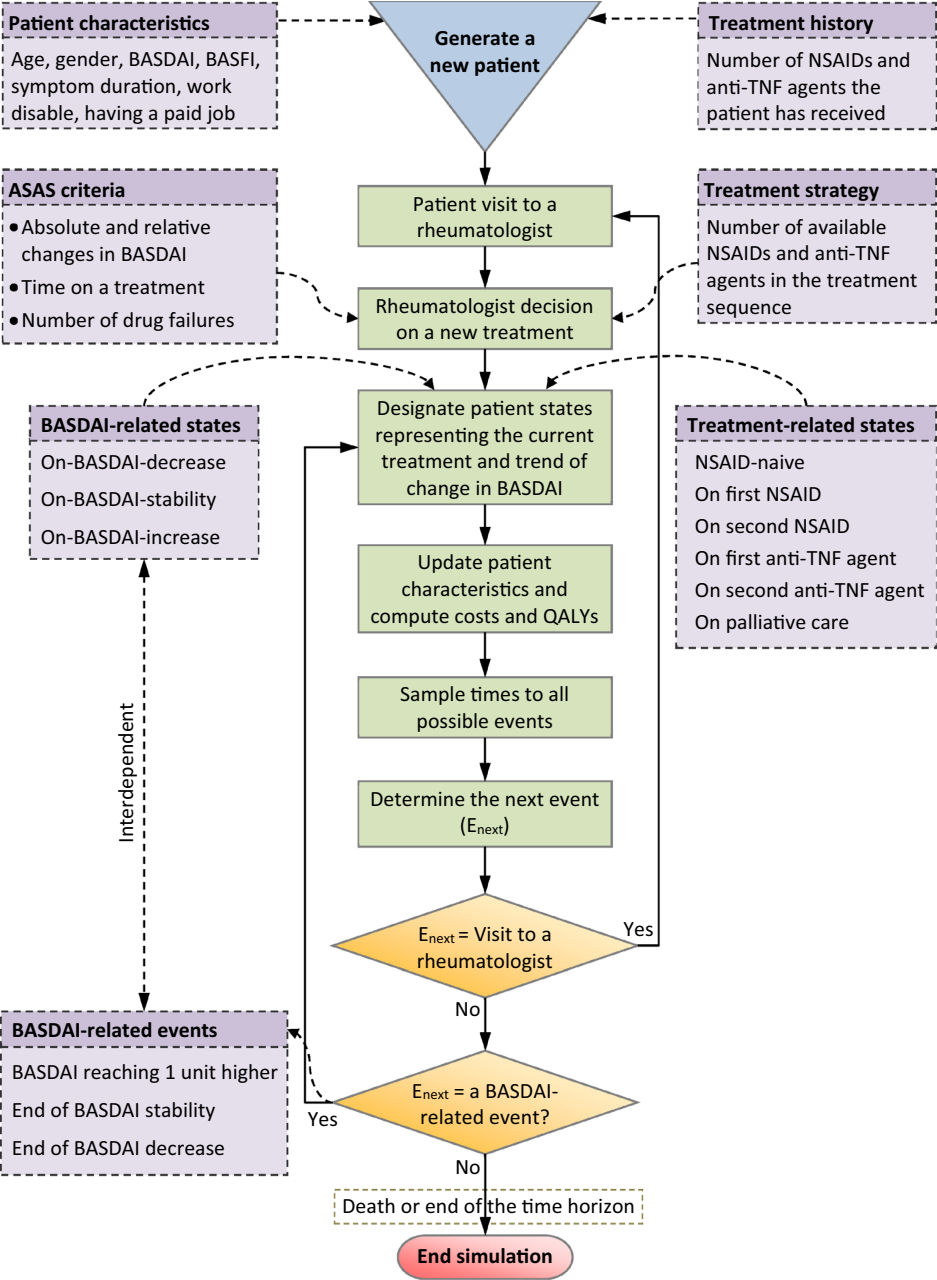
Main components and simulation process of the patient-level model used for tracking a patient with ankylosing spondylitis. For more information, see Tran-Duy et al. [20]

As in Tran-Duy et al. [20], the recommendations for treatment of AS from the Assessment in SpondyloArthritis International Society (ASAS) [9], which in many countries including The Netherlands have been adopted, were used to simulate the decision on starting and switching a drug based on BASDAI and the number of drugs that have been used. In the current prospective simulations, two treatment strategies were compared. In Strategy 1, anti-TNF agent would no longer be available from January 1, 2014 onwards; in Strategy 2, anti-TNF agents continued to be available. For *the prevalent AS population* on the starting date of the simulation, i.e. January 1, 2014, treatment history had to be simulated as there were no data available for the Dutch AS population on this date. Therefore, we performed two stages of simulation: (1) the burn-in stage in which a historical simulation was executed, starting from January 1, 1996 where data on the prevalent Dutch AS population were available, to obtain characteristics and treatment history of the prevalent population on January 1, 2014, and (2) the main stage in which prospective simulations were executed to quantify the AS population properties over time in different treatment strategies. For the *incident cases*, we assumed that no patients had received NSAIDs or anti-TNF agents before entering the simulation. Figure 1 conceptualises the main components and simulation process adapted from PLMod for use in the main stage of the current dynamic population model.

Opinion of rheumatologists were used to determine the maximal number of drugs in a sequential treatment strategy. In the historical simulation, a patient received a maximum of five NSAIDs during the life time, and a maximum of two anti-TNF agents from January 1, 2002, the date anti-TNF agents became reimbursed in The Netherlands, onwards. In the prospective simulations, a patient received a maximum of five NSAIDs in any strategy taking into account the number of NSAIDs already received in the burn-in stage; and received no more anti-TNF agent in Strategy 1, and a maximum of two anti-TNF agents in Strategy 2, taking into account the number of anti-TNF agents already received in the burn-in stage. In any simulation stage and treatment strategy, palliative care, i.e. the use of analgesics other than NSAIDs to alleviate symptoms, was given to the patient after failure of all the available drugs in the treatment. Algorithms for determining treatment failure and selecting a new treatment can be found in the study by Tran-Duy et al. [20] (Web Only Data Appendix 1: Details on the simulation process). Table 1 summarizes characteristics of the simulation stages and treatment strategies.

**Table 1:** Characteristics of the simulation stages and treatment strategies

	Burn-in stage (historical simulation)	Main stage (prospective simulation)	
	One treatment strategy	Treatment strategy 1	Treatment strategy 2
Time horizon	1 Jan 1996–1 Jan 2014	1 Jan 2014–1 Jan 2034	Same as treatment Strategy 1
Maximal number of drugs a patient can receive			
NSAIDs	Five, randomly selected from ten possible drugs After failure of two NSDAIs, the next two anti-TNF agents are considered; the remaining NSAIDs are reserved for those failing both anti-TNF agents	Five in an uninterrupted sequence, randomly selected from ten possible drugs	Same as in the burn-in stage
Anti-TNF agents	Two, one subcutaneous and another intravenous, available from 1 Jan 2002 onwards After failure of two anti-TNF agents, the remaining NSAIDs are considered	No longer available	Same as in the burn-in stage

*NSAIDs* non-steroidal anti-inflammatory drugs, *anti-TNF agent* tumour necrosis factor-α antagonist

### Dynamic population simulation

**Fig. 2:**
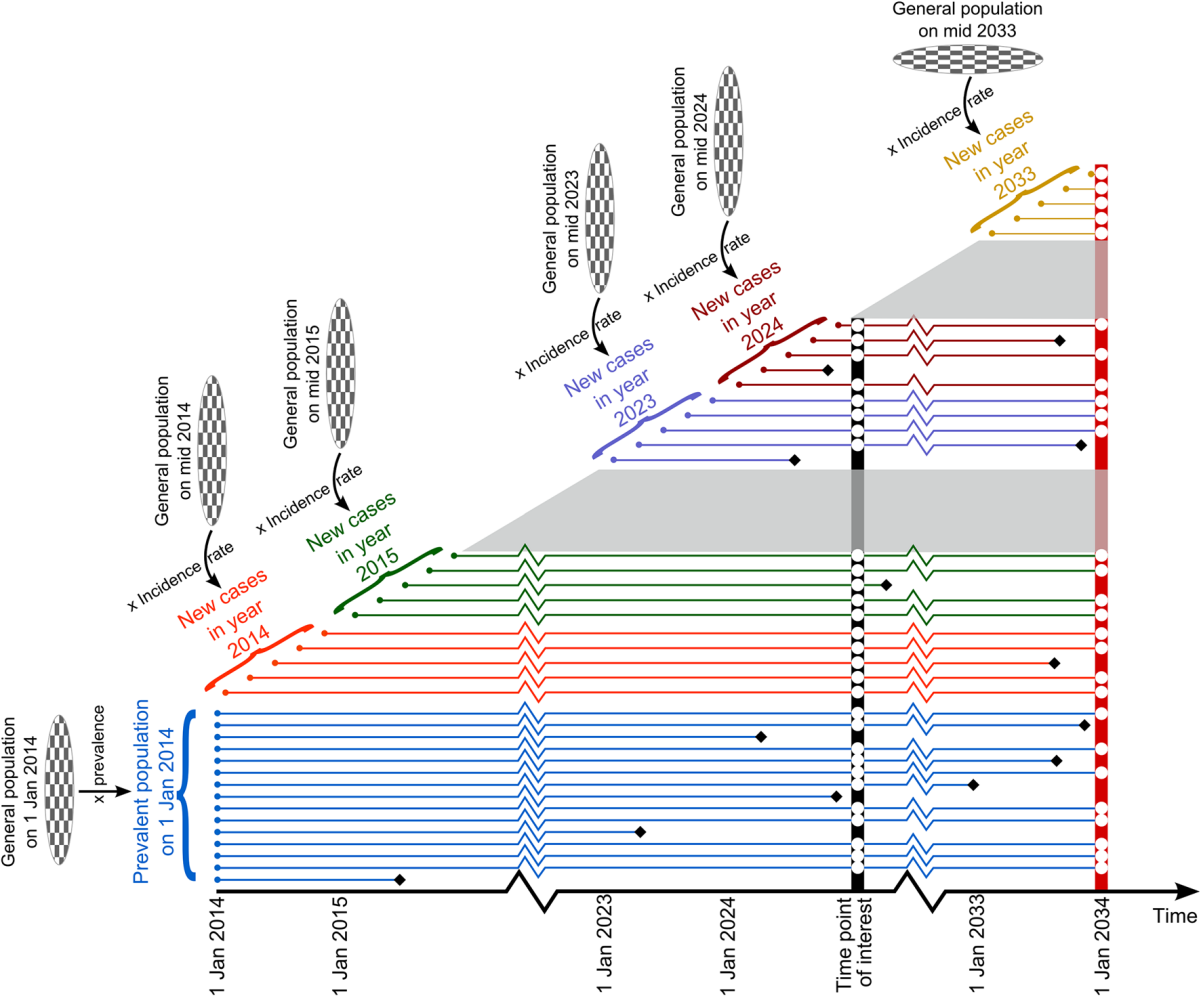
Conceptual model of the dynamic population simulation. Each *solid circular dot* on the *left* margin represents an AS patient at the time point when he or she enters the simulation. The patients are differentiated into groups, including the prevalent population on 1 January 2014 and incident cohorts in years 2014–2034, whose sizes are calculated based on the sizes of the general population and prevalence and incident rate. Each *solid horizontal line* represents the temporal movement of each patient, which ends when the patient dies (marked with a *solid diamond*) or reaches January 1, 2034 (marked with an *open circle* on the *vertical bar* on the right margin). The *open circles* represent the patients in the prevalent population at a time point of interest who are included in the summary statistics of disease measures, costs and health utilities

Given a decision time span of 20 years from January 1, 2014 to January 1, 2034 in the prospective simulations, the model tracked individually all AS patients appearing in The Netherlands during this period until death or end of the simulation time. The simulated patients comprised (1) the prevalent AS population (PP) on January 1, 2014, and (2) the yearly incident AS cohorts (ICs) in the subsequent years. The size of the PP was obtained from the historical simulation. The size of IC in each year was computed as the product of the size of mid general Dutch population in that year and the incidence rate of AS per year, which was assumed to be constant. To track the patients in each IC, a number of patients equalling the calculated size of the IC were created; for each patient, a set of characteristics were sampled based on appropriate probability distributions, which were assumed to be time-independent. For all the incident patients within a year, diagnosis duration was set at 0 when the patients enter the simulation assuming that a possible delay in diagnosis has no influence on the treatment selection or course of the disease. Figure 2 illustrates the concepts of the dynamic population simulation in the main stage. The same concepts were applied in the burn-in stage except for the start and end dates being January 1, 1996 and January 1, 2014, respectively, and the size of the PP being calculated as the product of the size of the general population on January 1, 1996 and the prevalence of AS, which was assumed to be constant.

### Model parameterization

We estimated probability distributions of the patient characteristics in the PP on January 1, 1996 and in the ICs using the data sources from the Outcomes Assessment in AS International Study (OASIS) [23] and expert opinion. Types and parameter estimates of the distributions of the characteristics are provided in Table 2. Since no recent data on incidence or prevalence of AS were available for The Netherlands, we set the AS prevalence at 0.4 %, as reported for Western countries in studies on the epidemiology of AS [24–26]. Studies in Spain, Finland and Northern Norway showed incident rates around 7 cases per 100,000 person-year [26–28]. We used this value as an initial point and calibrated it to obtain a mean prevalence of 0.4 % and a smallest variance of the simulated prevalence proportions at yearly time points. We derived the size of the general population on January 1 of each year and estimated mortality rate based on Statistics Netherlands [29]. We estimated drug efficacies based on literature, and costs and health utility (EQ-5D) using data from the OASIS [23]. A list of parameter estimates and detailed methods for parameter estimation are provided extensively in Tran-Duy et al. [20]. We assumed that the discontinuation of anti-TNF agents on Jan 1, 2014 in Strategy 1 caused a rebound of disease activity (BASDAI) to the baseline within 12 weeks based on expert opinion and observations in patients withdrawing from clinical trials [30].

**Table 2:** Types and parameter estimates of the distributions of the patient characteristics in the prevalent population on January 1, 1996 and in each yearly incident cohort

Characteristics	Distribution^a^	
	Prevalent population (PP) Estimated size: 62,000 patients	Yearly incident cohort Estimated size: 1000–1200 patients
Age of male	N (42.5, 12.6)	N (33.7, 8.14)
Age of female	N (46.3, 12.7)	N (33.7, 8.14)
Gender (male)	Bern (0.69)	Same as PP
Symptom duration	N (0.64 × Age − 7.76, 1.20 × $\sqrt {Age}$)^b^	Same as PP
Diagnosis duration	N (3.1, 1.3)^c^	0
Proportion of patients with paid jobs	Bern (0.75)	Same as PP
Proportion of paid-job patients being work disabled	Bern (0.20)	Same as PP
Contraindication to anti-TNF agents	Bern (0.20)	Same as PP
BASDAI	N (1.72, 0.63)^c^	N (1.91, 0.52)^c^
BASFI of male patient	N (0.69 × BASDAI + 1.89, 1.69)^d^	N (0.60 × BASDAI + 2.62, 1.42)^d^
BASFI of female patient	N (0.92 × BASDAI + 0.19, 1.00)^d^	N (0.91 × BASDAI + 0.27, 0.81)^d^

*BASDAI* Bath Ankylosing Spondylitis Disease Activity Index, *BASFI* Bath Ankylosing Spondylitis Functional Index

^a^N (m, s): Normal distribution with mean m and standard deviation s; Bern (p): Bernoulli distribution with probability of true value being p

^b^Distribution conditional on age; parameters estimated by fitting a linear model using generalized least squares with fixed variance weights

^c^Distribution of square root of the respective characteristics

^d^Distribution conditional on BASDAI

### Budget and health impact analyses

Costs incurred by and QALYs of each patient were computed for the period from the time point that the patient entered the simulation to death or January 1, 2034, whichever came first. Productivity costs were computed using the human capital approach [31]. Total population cost on January 1 of a specific year after 2014 was calculated as the sum of all costs due to AS in the period from January 1, 2014 to the respected year. Similarly, total population QALYs on January 1 of a specific year for the whole society were calculated as the sum of QALYs of all subjects having been diagnosed with AS by a rheumatologist between January 1, 2014 and the respected year. For probabilistic sensitivity analysis (PSA), values of the model parameters were sampled 10,000 times from the appropriate distributions; for each set of parameter values the simulation was run over the periods of 5, 10, 15 and 20 years starting from January 1, 2014. Net-benefit framework was used to construct the cost-effectiveness acceptability curves from the Monte Carlo simulation results [32].

### Modelling tools and model validation

We used the Delphi language (Embarcadero Delphi XE 2010, Embarcadero Technologies Inc, San Francisco, US) to program the dynamic population model, in which PLMod was integrated as an encapsulated module to generate outcomes of each patient from the model inputs. We used R [33] to analyse the simulated longitudinal outcomes of all individual patients. The model was rigorously checked for syntactical and logical errors. Simulated results were face-to-face validated by experts in AS and health economists.

## Results

### Predicted prevalent population on January 1, 2014

On January 1, 2014, the predicted numbers of male and female AS patients were 46,528 and 20,617, respectively; the simulated proportion of patients with paid jobs was 0.76, and proportion of patients with (partial) work disability was 0.19; the predicted numbers of patients receiving <2, 2 and >2 NSAIDs were 29,116, 7936 and 30,093; the predicted numbers of patients receiving 0, 1, and 2 anti-TNF agents were 37,467, 16,290 and 13,388. Summary statistics of the numeric characteristics of these patients are given in Table 3.

**Table 3:** Characteristics and treatment history of the simulated population of patients with ankylosing spondylitis on January 1, 2014

Attribute	Summary statistics of the 67,145 patients in the Dutch society based on the simulated data			
	Min	Median	Mean (SD)	Max
Age of male	15.6	43.2	43.8 (12.2)	80.5
Age of female	15.7	43.5	44.2 (12.3)	80.4
Symptom duration (year)	0	25.2	25.1 (10.8)	78.5
Diagnosis duration (year)	0	14.3	15.9 (10.7)	54.8
BASDAI (on 1–10 scale)	0	2.66	2.97 (1.57)	8.96
BASDFI (on 1–10 scale)	0	3.86	3.93 (1.92)	9.81

*BASDAI* Bath Ankylosing Spondylitis Disease Activity Index, *BASFI* Bath Ankylosing Spondylitis Functional Index

### AS population properties between January 1, 2014 and January 1, 2034

**Fig. 3:**
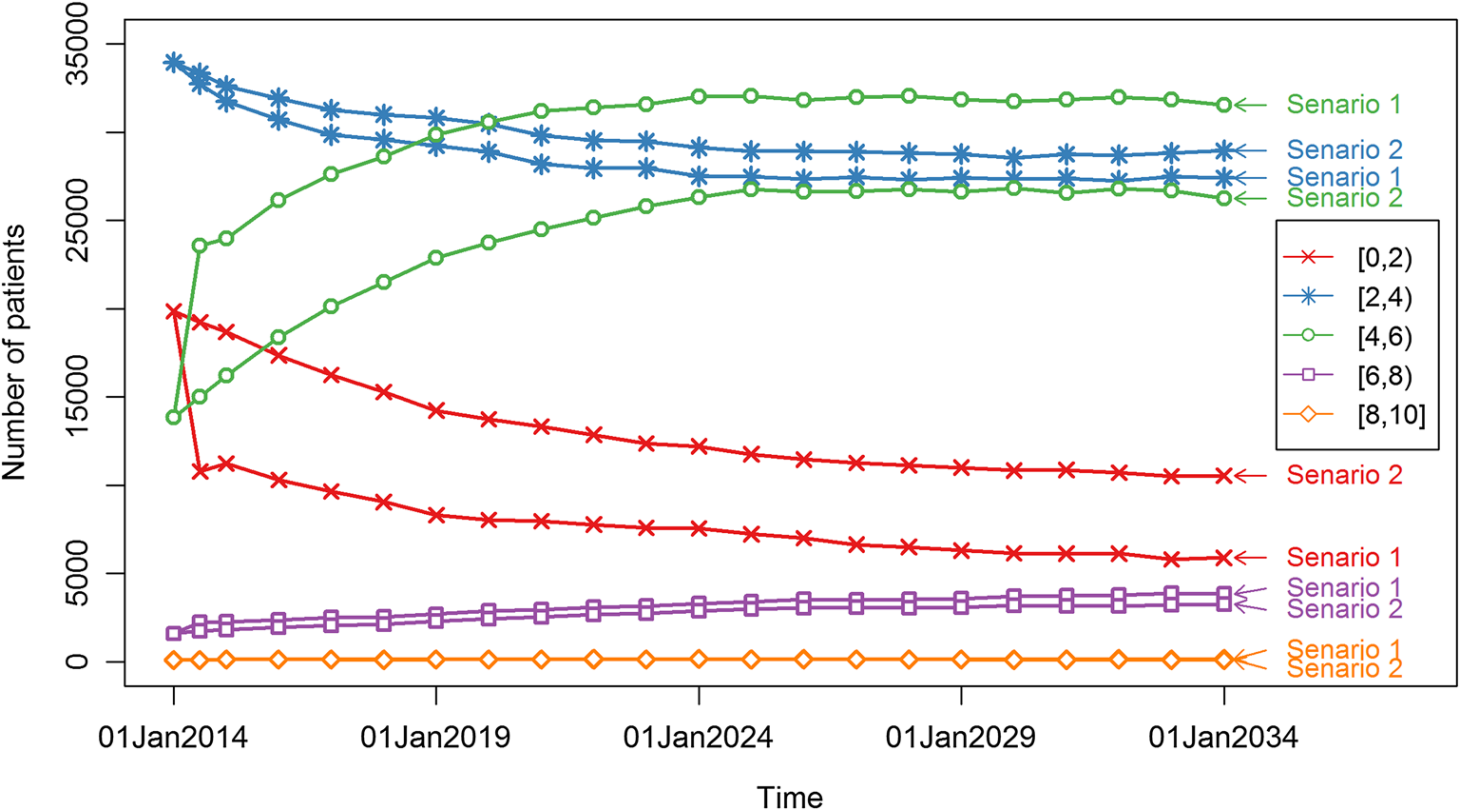
Changes over time in the number of patients with BASDAI within a specific interval in two treatment strategies. Strategy 1 consists of five available non-steroidal anti-inflammatory drugs (NSAIDs) and Strategy 2 consists of the same available NSAIDs as in Strategy 1 and two tumour necrosis factor-α antagonists

The predicted size of the prevalent AS populations at yearly time points between January 1, 2014 and January 1, 2034 varied within the range of 67,145–69,957, corresponding to a prevalence of approximately 0.4 %. Forty-four to 46 percent of the patients received anti-TNF agents over the period from 2014 to 2034. The numbers of AS patients with low BASDAI in the intervals [0, 2) and [2, 4) were higher in Strategy 2 than in Strategy 1, while those with moderate to high BASDAI in the intervals [4, 6) and [6, 8) were higher in Strategy 1 than in Strategy 2 over time (Fig. 3; the square bracket or the parenthesis at one end of an interval indicates that its adjacent endpoint is included or excluded, respectively). Differences in the number of AS patients with very high BASDAI in the interval [8, 10] were negligible between the two strategies. Inclusion of two anti-TNF agents in the AS treatment (Strategy 2) affects the numbers of AS patients with BASDAI in the intervals [4, 6) and [0, 2] more pronouncedly in the other intervals.

Changes over time in mean BASDAI and BASFI of the AS population in both strategies followed the same trends. When only NSAIDs but no anti-TNF agent were available after January 1, 2014 (Strategy 1), mean BASDAI and BASFI over 12 weeks increased from 2.97 to 3.55, and from 3.93 to 4.45, respectively. After March 2014, mean BASDAI and BASFI slightly increased during the next 10 years and then remained almost constant during the period from 2024 to 2034 at 3.87 and 4.91, respectively. Ranges of means (SDs) of BASDAI and BASFI at yearly time points over 20 years were 2.97–3.89 (1.51–1.59), and 3.93–4.94 (1.86–1.95), respectively.

When two anti-TNF agents remained available after January 1, 2014 (Strategy 2), there was almost no change in mean BASDAI and BASFI over time; means (SDs) of BASDAI and BASFI at yearly time points between January 1, 2014 and January 1, 2034 ranged from 2.97 to 3.13 (1.53–1.61), and from 3.93 to 4.12 (1.85–1.93), respectively.

In line with changes in mean BASDAI and BASFI, mean health utility quickly decreased in Strategy 1 from 0.59 to 0.52 within 3 months, after which it slightly decreased during the next 10 years and then remained almost constant at 0.48 until 2034. In Strategy 2 mean health utility remained almost constant at 0.59 over the period from January 1, 2014 to January 1, 2034.

### Budget and health impacts, and incremental cost-effectiveness

Cumulative societal total population costs (in million Euros) incurred by all actual AS patients in the Dutch Society from January 1, 2014 onwards, based on the current prices assuming no inflation or major change in treatment options, on January 1 of 2019, 2024, 2029 and 2034 would be 3412.03, 6786.76, 10,139.94 and 13,481.54 in Strategy 1, respectively, and 4113.66, 8126.46, 12,089.74 and 16,022.27 in Strategy 2, respectively. Total annual direct and productivity costs (in million Euros) in Strategy 1 ranged from 290.58 to 326.02, and from 344.86 to 381.05, respectively. In Strategy 2, total annual direct and productivity costs (in million Euros) ranged from 470.61 to 496.88, and from 323.05 to 354.95, respectively. The use of anti-TNF agents resulted in an increase in the annual drug costs (€168.54–205.28 million Euros), but at the same time caused a decrease in the annual productivity costs (€12.58–31.21 million Euros) and in annual costs of healthcare categories other than drugs (€7.23–11.90 million Euros).

Cumulative QALYs of all actual AS patients in the Dutch society since January 1, 2014 on January 1 of 2019, 2024, 2029 and 2034 would be 239,174, 462,920, 687,465 and 903,983, respectively, in Strategy 1, and would be 248,482, 482,831, 717,960 and 94,5546, respectively, in Strategy 2. The use of anti-TNF agents resulted in an increase in the annual total QALYs of 1628–2206.

Incremental cost (€) per QALY gained (iCER) in Strategy 2 compared with Strategy 1 on January 1 of 2019, 2024, 2029 and 2034 improved slightly from 75,379 to 67,268, 63,938 and 61,129, respectively.

### Probabilistic sensitivity analysis

**Fig. 4:**
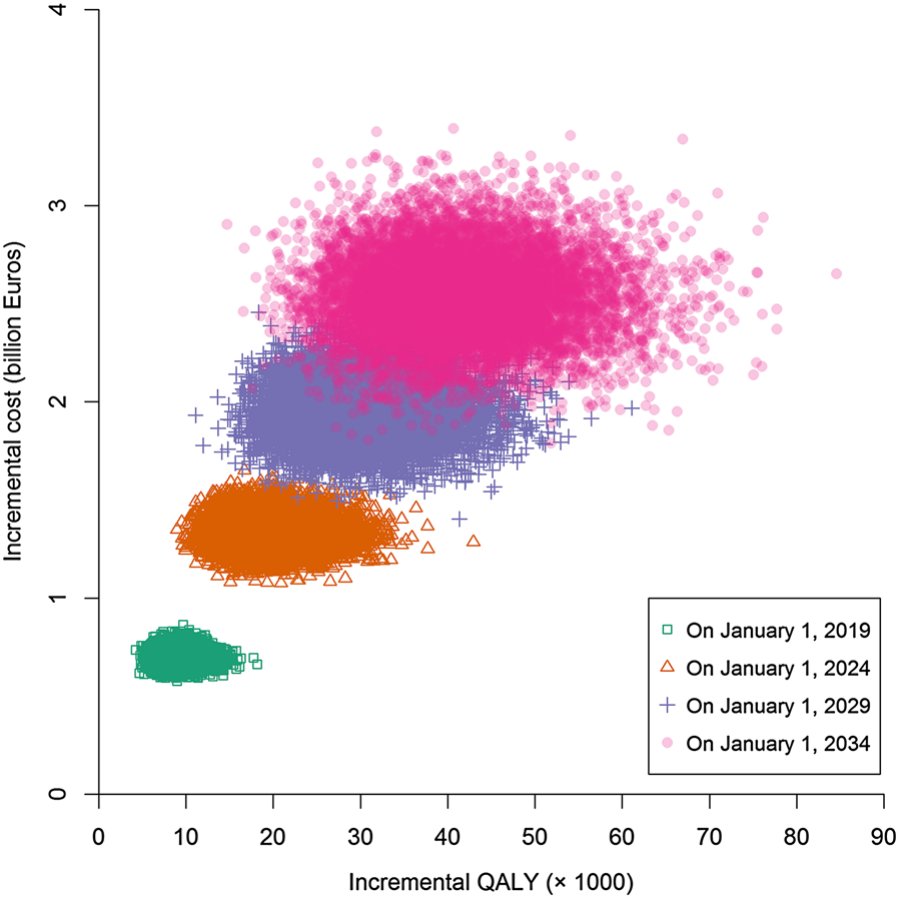
Scatter plot of incremental total population costs against incremental total population quality-adjusted life year (QALYs) in Strategy 2 (alternative) compared to Strategy 1 (reference). Strategy 1 consists of five available non-steroidal anti-inflammatory drugs (NSAIDs) and Strategy 2 consists of the same available NSAIDs as in Strategy1 and two tumour necrosis factor-α antagonists. The clouds corresponding to January 1 of 2019, 2024, 2029 and 2034 were obtained from simulations with four different time spans of decision, 5, 10, 15 and 20 years, respectively. In each cloud, each data point was obtained from one run of simulation for all the AS patients appearing in the period from January 1, 2014 to the end of the corresponding time span with a set of model parameter values sampled from appropriate probability distributions; 10,000 runs were executed which resulted in 10,000 data points. All the data points in the four clouds lie in the north-east quadrant of the plane

**Fig. 5:**
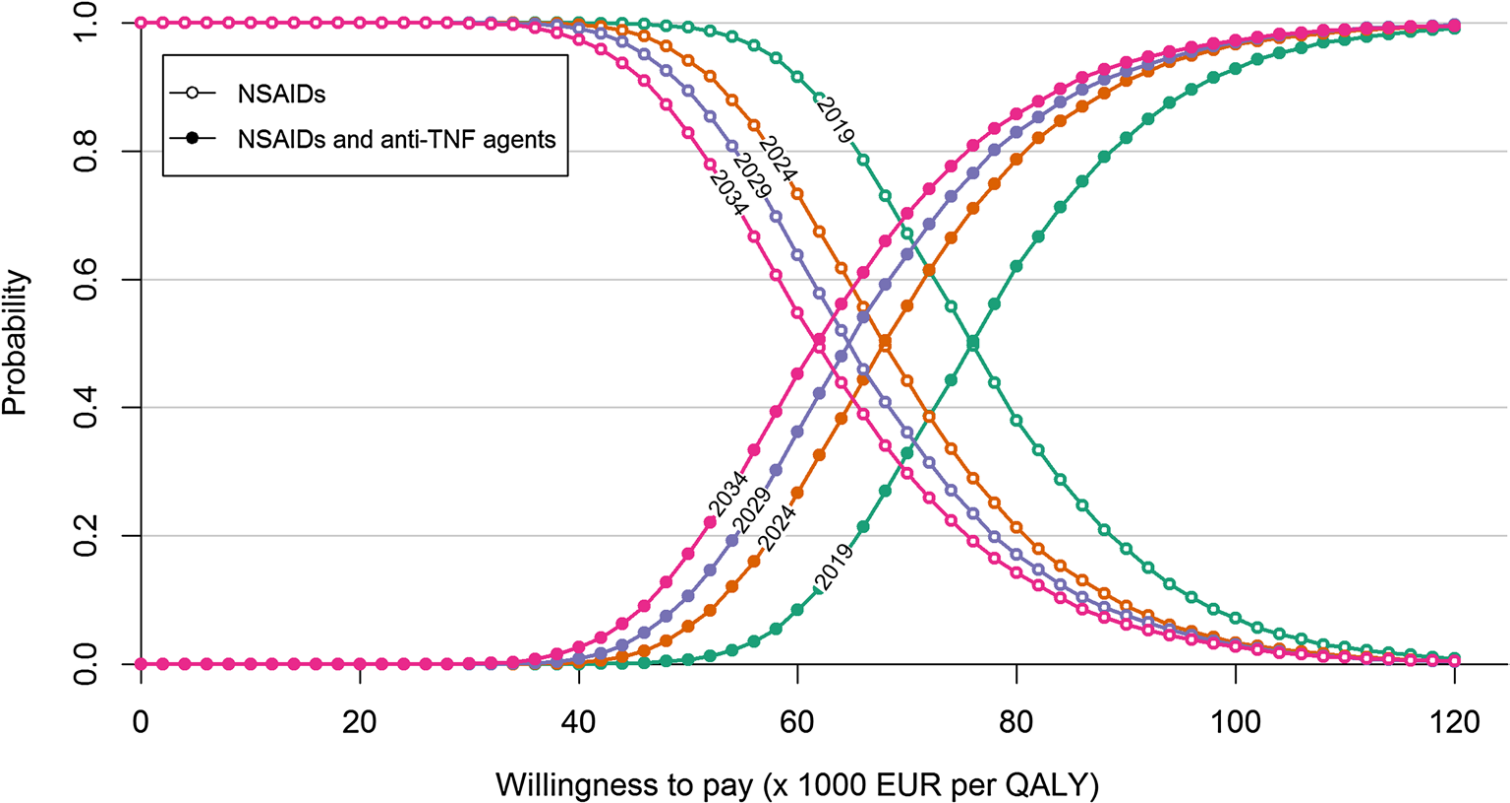
Cost-effectiveness acceptability curves for two treatment strategies based on the uncertainty in cost and quality-adjusted life year (QALY) on January 1 of 2019, 2024, 2029 and 2034. Strategy 1 consists of five available non-steroidal anti-inflammatory drugs (NSAIDs) and Strategy 2 consists of the same available NSAIDs as in Strategy 1 and two tumour necrosis factor-α antagonists (anti-TNF agents). The *four curves* were obtained from simulations with four different time spans of decision, 5, 10, 15 and 20 years

The scatterplot of the joint uncertainty in the incremental total population costs against incremental total population QALYs on January 1 of 2019, 2024, 2029 and 2034 in Strategy 2 compared with Strategy 1 showed all data points lying within the north-east quadrant of the cost-effectiveness plane (Fig. 4). The boundaries of incremental cost and QALY and the ranges of incremental cost and QALY increased with increasing time horizons. At willingness-to-pay amounts for one QALY gain (WTPs) of €75,870, €67,885, €64,663 and €61,783, the probabilities that Strategy 1 and Strategy 2 are cost-effective were equal (0.5) on January 1 of 2019, 2024, 2029 and 2034, respectively. At WTPs of €118,656, €112,067, €110,188 and €110,512, it was 99 % certain that Strategy 2 was cost-effective on January 1 of 2019, 2024, 2029 and 2034, respectively (Fig. 5).

## Discussion

Budget impact analysis (BIA) is increasingly becoming a required part of economic evaluation of a new health intervention [34]. In the Netherlands and many other countries like England, USA and Australia, reimbursement of a new drug is based not only on CE information but also on an additional appraisal that includes societal cost impact of the drug treatment on the national health and healthcare budget [14, 35]. However, in most studies the BIA has been carried out independently of health impact analysis and analysis of the actual incremental CE. As a consequence, the information provided has usually been incomplete for a healthcare system decision that takes the outcomes of any patients receiving the intervention into consideration. In the present study we used a state-of-the art approach to quantify the total population health and societal cost associated different treatment strategies for AS, and derived the actual iCER of the treatment including anti-TNF agents compared with usual care for different time spans of decision. In AS, so far the CE of anti-TNF agents reported in the literature has been analysed using a closed cohort of patient with arbitrary initial sizes. Except for the study by Tran-Duy et al. [20], all the studies compared a single anti-TNF agent with usual care and reported iCERs for infliximab, etanercept and adalimumab being in the ranges of €5307–€237,010, €29,815–€123,761 and €7344–€33,303, respectively [19]. In the study by Tran-Duy et al. [20], CE of sequential treatment strategies rather than single drugs were modelled and the computed iCER of the treatment strategy that included both NSAIDs and anti-TNF agents compared to the treatment strategy that included only NSAIDs was €35,186. Although the present model adopted the same setting for comparators and the same equations for computing costs and QALYs, its predicted iCERs were higher than the iCER in Tran-Duy et al. [20]. This difference in iCERs was due to the difference in the patient populations considered in the models. In Tran-Duy et al. [20], a closed cohort of 13,000 newly diagnosed cases, determined based exclusively on technical aspects, was used for the simulation, and the size of this cohort decreased over time. In contrast, the present model started with a realistic prevalent population of 67,145 patients, and the size of the prevalent population slightly increased over time as a result of increased sizes of the incident cohorts who entered the simulation over time. The effect of incorporating population dynamics on the model outcomes can easily be seen via changes in disease measures. After 6 months since the start of the simulation, the simulated mean BASDAI and BASFI in Tran-Duy et al. [20] increased over time, but those in the current study were quite stable. This implies that incorporating the population dynamics in the model affects both the incremental cost and incremental QALYs of the treatment strategy with anti-TNF agents, and therefore iCERs, because BASDAI and BASFI influence resource utilisation and health utility.

The difference in the predicted iCER between the closed cohort model in Tran-Duy et al. [20] and the dynamic population model in the present study is in line with the findings of Hoyle and Anderson [36]. Using algebraic expressions, the authors of that study [36] showed that the iCER computed by a conventional CE analysis may substantially differ from that by the model that includes both prevalent and incident cohorts, especially when the discount rates for costs and QALYs are unequal. In The Netherlands, the annual discount rates for costs of 4.0 % and for QALY of 1.5 % are recommended [35], which were used in both our previous [20] and present models. The findings from our studies argue in favour of the suggestion of Hoyle and Anderson [36] that, to inform a decision that affects costs and benefits of all patients in the healthcare system, a CE analysis should include both prevalent and future incident patient cohorts.

Despite the difference in the modelling approach, both study by Tran-Duy et al. [20] and the current study showed that inclusion of anti-TNF agents in the sequential treatment of AS would be cost-effective in The Netherlands, where the maximal acceptable willingness-to-pay (WTP) threshold is suggested to be around €80,000 per QALY [37]. The findings from the present study also indicate that given a WTP threshold, the certainty in decision on the CE of using anti-TNF agents in the treatment of AS is influenced by the time frame of the assessment. For example, while it was 50 % certain that the use of anti-TNF agents over 20 years was cost-effective, it was only 10 % certain that the use of anti-TNF agents over 5 years was cost-effective, given a WTP threshold of €61,783 per QALY.

We showed that retaining anti-TNF agents in management of AS would result in substantial gain in health for the population compared with the treatment strategy that withdraws anti-TNF agents. In addition, 10 % of the increase in the annual medication expenditures due to the use of anti-TNF agents would be compensated by the financial return from increased productivity and reduced need for hospitalisations. By partitioning the costs into different categories and computing them at different time points after the introduction of the new treatment option in real life, the model results can be used to better inform decisions on organisation of healthcare resources at the regional and/or national level.

In a dynamic population model, changes in the patient population properties over time are influenced by the incidence and mortality rates, and the distributions of patient characteristics of the initial prevalent population and of the subsequent incident cohorts. A literature review showed that these data in AS are still scarce [38], which suggests that a thorough study on demography and epidemiology of the AS population in the modelled society should be conducted. We assumed that prevalence and incidence rate were constant but in reality they may decrease over time with better prevention and treatments. Predicting the trend of changes in these quantities is challenging; therefore, it is worthwhile to analyse scenarios with different trends of change in prevalence and incident rate in a dynamic population model.

## Conclusions

Using the dynamic population approach, our model can project real-time data to inform a healthcare system decision that affects all actual number of AS patients eligible for anti-TNF agents within different time spans of decision. The predicted total population costs of different categories in the present study can help plan the organization of the healthcare resources based on the national budget for the disease. The use of anti-TNF agents in a dynamic population would be marginally acceptable in The Netherlands. Our study showed the knowledge gaps with regards to the prevalence and incident rate and the distributions of the patient characteristics in the prevalent population and incident cases, suggesting a need for conducting relevant studies to fill these gaps.

## Abbreviations

Anti-TNF agent: tumour necrosis factor-α antagonist
AS: ankylosing spondylitis
ASAS: Assessment in SpondyloArthritis International Society
BASDAI: Bath Ankylosing Spondylitis Disease Activity Index
BASFI: Bath Ankylosing Spondylitis Functional Index
BIA: budget impact analysis
CE: cost-effectiveness
CEA: cost-effectiveness analysis
IC: incident cohort
iCER: incremental cost-effectiveness ratio
NSAID: nonsteroidal anti-inflammatory drug
OASIS: Outcomes Assessment in Ankylosing Spondylitis International Study
PLMod: patient-level model
PP: prevalent population
PSA: probabilistic sensitivity analysis
QALY: quality adjusted life year
WTP: willingness-to-pay
